# Physical Performance Tests Correlate With Patient-reported Outcomes After Periacetabular Osteotomy: A Prospective Study

**DOI:** 10.5435/JAAOSGlobal-D-21-00100

**Published:** 2021-06-08

**Authors:** Elizabeth J. Scott, Michael C. Willey, John C. Davison, Robert W. Westermann, Amanda C. Paulson, Jason M. Wilken

**Affiliations:** From the Department of Orthopedics and Rehabilitation, University of Iowa Hospitals and Clinics, Iowa City, IA.

## Abstract

**Introduction:**

Individuals with hip dysplasia report significant functional disability that improves with periacetabular osteotomy (PAO). Four physical performance measures (PPMs) have been recently validated for use with nonarthritic hip conditions; however, their ability to detect functional improvement and correlate with improvements in popular hip-specific patient-reported outcome (PRO) instruments after PAO is unknown. The purpose of this study was to evaluate the responsiveness of four PPMs up to 1 year after PAO, compare PPMs with established PRO measures at these time points, and report the acceptability and utility of PPMs for assessing outcomes after PAO.

**Methods:**

Twenty-two participants aged 15 to 39 years completed the timed stair ascent (TSA), sit-to-stand five times (STS5), self-selected walking speed, four-square-step test, and seven hip-specific PRO measures before surgery and at approximately 6 months and 1 year after PAO. They completed questions regarding acceptability and utility of both types of testing. Wilcoxon rank sum test and unpaired Student *t*-tests were used to assess differences between time points; Spearman correlation and generalized linear modeling were used to determine the relationship between PPMs and PRO measures.

**Results:**

Six months after PAO, participants showed significant improvements on all seven PRO instruments (*P* < 0.001) and on the STS5 (*P* = 0.01). At one year, these improvements were maintained and TSA also improved (*P* = 0.03). Improvement in other PPMs did not reach significance (*P* = 0.07 and 0.08). The STS5 test demonstrated moderate to strong correlation (|r| = 0.43 to 0.76, *P* < 0.05) with all PRO measures, and the TSA test demonstrated moderate to strong correlation with almost all measures (|r| = 0.43 to 0.58, *P* < 0.05). Correlations strengthened on subanalysis of participants with unilateral disease (n = 11) (|r| = 0.56 to 0.94, *P* < 0.05). All participants (100%) found PPM testing acceptable despite disability; 25% preferred PPMs to PRO measures, whereas 75% of participants found them equal in usefulness.

**Discussion:**

The STS5 and TSA tests demonstrated moderate to very strong correlation with PRO measures at six and 12 months after PAO for dysplasia. These tests could be used as a functional outcome to supplement PRO instruments after PAO.

Periacetabular osteotomy (PAO) is a well-established surgical procedure to treat acetabular dysplasia in the skeletally mature, nonarthritic hip.^1,2,3,4^ The typical patient is young and active with expectation for return to a high level of function after treatment. Measuring functional deficit is typically done with hip-specific patient-reported outcome (PRO) instruments such as the hip disability osteoarthritis outcome score (HOOS),^8^ International Hip Outcome Tool (iHOT),^5^ modified Harris hip score (mHHS), Western Ontario and McMaster Universities Osteoarthritis Index,^6^ or Patient-Reported Outcome Measurement Information System Physical Function (PROMIS PF).^7^ Although these tools are validated for use in hip preservation surgery and correlate well with one another after PAO,^8,9,10,11^ PRO instruments can impose substantial test burden and are limited by their reliance on patient recall and self-perception.^12,13^

Physical performance measures (PPMs) allow objective assessment of impairment and recovery and provide information complementary to PROs.^14-16^ Performance-based outcome measures are gaining widespread use to assess recovery after athletic injury and to evaluate the effects of hip and knee osteoarthritis.^17,18,19,20^ The use of physical performance measures after surgical treatment of nonarthritic hip conditions is not widely reported.^21^

Four PPMs have been recently explored for use with both hip impingement and dysplasia to correlate with common PRO measures:^22,23^ the sit-to-stand five times (STS5) test, four-square-step test (FSST), self-selected walking speed (SSWS), and timed stair ascent (TSA).^8,19^ Participants with symptomatic hip dysplasia demonstrate disability with slower time to completion or walking speed on all four tests compared with healthy peer subjects.^22^ The utility of these tests in the postoperative setting has not been explored. The purpose of this study was to (1) evaluate the responsiveness of these four PPMs to at 6 months and one year after PAO, (2) compare these PPMs with established hip-specific PRO measures, and (3) report the acceptability and perceived benefit by patients in assessing postoperative outcomes. We hypothesized that (1) participants would show and maintain significant improvement on all four PPMs after PAO at 6 months and 1 year, (2) PPMs would correlate highly with function-based PRO measures, and (3) participants would find PPM testing acceptable to perform and more useful than PRO instruments.

## Methods

This prospective study was approved by our institutional review board. All participants were enrolled at a single institution. Patients aged 15 to 39 years who were indicated for PAO surgery during the 8-month enrollment period (May 2018 to January 2019) were eligible for inclusion. Exclusion criteria included previous ipsilateral femoral or pelvic osteotomy, neuromuscular condition, history of Perthes disease, or slipped capital femoral epiphysis. Participants were compensated up to $100.00 each over the course of the study.

### Preoperative Workup

Standing AP radiographs were used to assess lateral center-edge angle (LCEA) of Wiberg, Tönnis angle, extrusion index, and Tönnis grade. Alpha angle was measured on Dunn lateral and frog-leg lateral views. All measurements were done by a fellowship-trained surgeon (M.C.W.). PAO was indicated for patients who presented to clinic with hip pain, LCEA less than 20° or LCEA 20°—25° with hypermobility, Tönnis grade 0 or 1, and failure of nonoperative treatments including physical therapy, activity modification, and intra-articular steroid injections. Hip arthroscopy in addition to PAO was indicated when there was labral injury or cartilaginous pathology on hip MRI or when there was a history of previous hip arthroscopy.

### Outcomes Assessment

**Figure 1:**
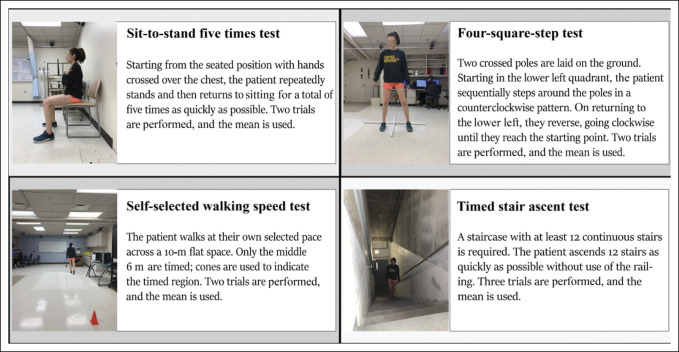
Illustration showing the four physical performance measures used in this study. Participants completed the tests in the same order at each visit.

PROs and PPMs were collected at four separate study visits: two preoperative visits staged at least 24 hours apart and postoperative visits at 6 months and 1 year. This study used data from the first preoperative visit only; the second preoperative visit was used in a previous study for interrater (Intraclass Correlation Coefficient [ICC] 0.97 to 0.99) and intrarater (ICC 0.83 to 0.93) reliability testing.^22^ At each assessment, participants completed seven PRO instruments: visual analog scale (VAS) for pain, International Hip Outcome Tool short version (iHOT-12),^5^ hip disability and osteoarthritis outcome score short version (HOOS PS)^24^ and pain subscale (HOOS Pain),^10^ PROMIS physical function and pain interference adaptive tests (PROMIS PF and PROMIS PI),^25,26^ and modified Harris hip score (mHHS).^27^ PRO questionnaires were administered in a randomized order using a handheld tablet computer. Participants were also asked to report frequency of opioid use in the past 30 days. After administration of PRO instruments, the participants proceeded to functional testing with a trained examiner (J.D.) (Figure 1). The PPM standardized protocol has been previously described.^22^

After performance testing, participants completed an electronic survey assessing (1) perceived difficulty and acceptability of the PPMs, (2) perceived performance compared with previous visits, and (3) how the PPM testing compared in utility and difficulty with PRO testing.

### Statistical Analysis

All variables were evaluated for normality, and nonparametric methods were used when indicated. For all numeric variables, mean, median, minimum and maximum, standard deviation, and range were calculated. Wilcoxon rank sum test was used to compare PPMs and PRO measures between each data collection; to account for variation in follow-up time points between participants, linear mixed models were used to assess for changes in scores over time, with *P* values adjusted for multiple comparisons. Unpaired Student *t*-tests (alpha = 0.5) or Wilcoxon rank sum test where appropriate was used to compare body mass index (BMI), age, and radiographic data. Fisher exact test was used for comparison of categorical variables including opioid use and sex.

Spearman rank correlations were used to determine the relationship between the PPMs and PRO measures at each time point. Correlations were defined as very strong (*r* > 0.7), strong (*r* = 0.61 to 0.69), moderate (*r* = 0.4 to 0.6), moderately weak (*r* = 0.31 to 0.39), and weak (≤0.3). Statistical analysis was done by a trained statistician using SAS software (SAS version 9.4; SAS Institute).^28^ Statistical significance was considered *P* < 0.05, and Bonferroni-Holm correction was used to correct for multiple comparisons.

## Results

### Demographics

**Figure 2:**
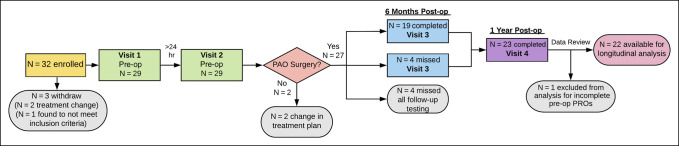
Flow diagram depicting study follow-up.

Thirty-two individuals were enrolled, and 27 of the 32 participants underwent PAO surgery. Of these 27 patients, 22 completed both preoperative and postoperative PRO and PPM data collection and were included in the full statistical analysis (Figure 2). Most participants were female patients (20/22), and half (11/22) had bilateral hip pain. The 6-month follow-up occurred at an average of 6.3 ± 0.9 months after surgery with 70% completion rate and the 1-year follow-up at an average of 12.9 ± 1.9 months after surgery with 81% completion rate. Subject demographic and radiographic data are detailed in Table 1. One participant had undergone previous hip arthroscopy. Most participants (18/22) had a concomitant arthroscopy at the time of PAO, which included femoral offset correction (n = 18), labral repair (N = 15), subspine decompression (n = 3), and labral reconstruction (n = 1).

**Table 1:** Demographics and Radiographic Values for Participants Included in Longitudinal Analysis (N = 22)

Variable^a^	Pre-op n = 22	6 Months Post-PAO n = 19	12 Months Post-PAO n = 22	*P*^b^ 0–6 mo n = 19	*P*^b^ Δ0-12 mos n = 22
Age, yr	24.5 ± 9.3	24.9 ± 9.2	25.5 ± 9.1	NS	NS
Body mass index	23.7 ± 4.0	25.2 ± 7.1	24.6 ± 4.7	NS	NS
Female sex, n (%)	20 (90.9)	17 (89.4)	21 (91.3)	NS	NS
Opioid use, n (%)					
None	17 (77.2)	19 (100)	21 (95.4)	0.068	0.078
Intermittent	4 (18.1)	0	0		
Daily	1 (4.55)	0	1 (4.3)		
LCEA, °	18.0 ± 7.0 (6 to 41)	36.1 ± 5.6 (31 to 44)	37.4 ± 3.5 (31 to 46)	<0.0001	<0.0001
Tönnis angle	12.4 ± 5.9 (0.0 to 27.0)	0.1 ± 3.9 (−5.0-5.2)	−0.8 ± 3.4 (−8.0-5.2)	<0.0001	<0.0001
Extrusion index	0.3 ± 0.0 (0.13 to 0.43)	0.1 ± 0.0 (0.08 to 0.20)	0.1 ± 0.0 (0.04 to 0.20)	<0.0001	<0.0001
Posterior wall sign (n/n)	6/22	0/19	0/22	0.010	<0.0001
Alpha angle, °	60.4 ± 7.3 (44 to 72)	44.7 ± 5.2 (36 to 57)	44.3 ± 5.4 (36 to 57)	<0.0001	<0.0001
Femoral version, °	14.6 ± 13.2 (−6.0-46.0)	12.3 ± 11.3 (−6.0-30.5)	14.6 ± 13.2 (−6.0-46.0)	NS	NS
Tönnis grade (n)	(18) Tönnis 0 (4) Tönnis 1	(16) Tönnis 0 (3) Tönnis 1	(18) Tönnis 0 (3) Tönnis 1 (1) Tönnis 2	NS	NS
Laterality of surgery, n (%)					
Unilateral	11 (50)	10 (52)	11 (50)	NS	NS
Bilateral	11 (50)	9 (47)	11 (50)		
Concomitant arthroscopy with PAO, n (%)	18 (81)				
Subsequent ipsilateral Procedures, n	—	2 ROI	20 ROI		
Subsequent contralateral procedures, n	—	None	6 PAO + scope 1 scope only 1 ROI		

LCEA = lateral center-edge angle, NS = not significant, PAO = periacetabular osteotomy, ROI = removal of implant

a All values expressed as mean ± standard deviation (range) unless otherwise specified. The LCEA was measured on coronal CT. The extrusion index, Tönnis angle, Tönnis grade, and posterior wall sign were measured on anteroposterior standing radiograph.

b Fisher exact test used for opioid use; otherwise, unpaired Student *t*-test or Wilcoxon rank sum was used where appropriate.

Complications after surgery included one superior ramus nonunion with persistent pain, which was treated with open reduction and internal fixation 18 months postoperatively. All but two participants (N = 20/22) underwent removal of implant between the 6-month and 1-year follow-ups. Seven patients with bilateral hip pain also underwent arthroscopic or open surgery on the contralateral hip during the follow-up, including arthroscopic labral repair with capsular plication (n = 1), capsular débridement (n = 1), PAO ± arthroscopy (n = 6), and implant removal (n = 1).

### Patient-Reported Outcomes

Scores for all PRO measures improved significantly at 6 months (all *P* ≤ 0.0002), with some cases reaching the level of healthy control subjects of similar age and sex^22^ (Table 2). Scores at 6 months and 1 year were not significantly different (all comparisons *P* > 0.05). For PRO measures with an available minimal clinically important difference (MCID) (iHOT-12,^29^ HOOS Pain,^8^ HOOS PS,^30^ and mHHS^8^), 86.3 to 94.7% of participants improved by at least the MCID, and the mean change in score for all participants was more than three times the MCID at both follow-ups (Tables 2 and 3). When comparing participants with bilateral and unilateral dysplasia, no significant difference was observed in the percentage of participants who attained MCID at six or 12 months. Finally, on generalized linear modeling, PAO had a strong effect on all PRO measures at the 6-month and 1-year follow-ups (all *P* < 0.0001 with correction for multiple comparisons; see supplemental Table 1, http://links.lww.com/JG9/A138).

**Table 2:** PROs of Participants at Baseline, 6 Months, and 1 Year After PAO Surgery

PRO Instrument	Pre-PAO n = 22^a^	6 mo Post-PAO n = 19	12 mo Post-PAO n = 22	Δ0-6 mo Post-PAO^b^ n = 19	Δ0-12 mo Post-PAO^b^ n = 22
VAS	55.2 ± 21.8 10.0 to 80.0	11.4 ± 15.4 0.0 to 57.0	19.5 ± 25.7 0.0 to 81.0	−42.2 ± 22.7 −80.0 to 2.0 *P ≤ 0.0001*	−35.7 ± 26.1 −80.0 to 4.0 *P ≤ 0.0001*
HOOS pain	47.4 ± 14.3 27.5 to 77.5	85.7 ± 17.0 45.0 to 100.0	82.3 ± 24.4 7.5 to 100.0	36.4 ± 15.7 10.0 to 67.5 *P ≤ 0.0001*	34.9 ± 22.2 −32.5 to 67.5 *P ≤ 0.0001*
HOOS PS	38.5 ± 15.7 8.8 to 74.8	81.8 ± 23.3 23.4 to 100	78.8 ± 26.4 16.4 to 100	40.9 ± 22.2 0.0 to 73.1 *P ≤ 0.0001*	40.3 ± 23.3 −21.3 to 73.1 *P ≤ 0.0001*
iHOT-12	31.9 ± 12.7 12.8 to 58.0	80.7 ± 20.5 25.7 to 100	76.2 ± 27.5 14.1 to 100	48.4 ± 19.5 8.1 to 79.7 *P ≤ 0.0001*	44.3 ± 24.6 −20.0 to 79.6 *P ≤ 0.0001*
PROMIS PF *t*-score	41.3 ± 6.2 34.7 to 61.7	52.3 ± 8.4 34.7 to 73.3	52.2 ± 9.8 34.7 to 73.3	10.8 ± 6.8 −3.4 to 24.6 *P ≤ 0.0001*	11.2 ± 9.8 −9.0 to 36.2 *P ≤ 0.0001*
PROMIS PI *t*-score	61.8 ± 4.5 51.9 to 68.4	46.6 ± 8.3 38.7 to 64.2	46.9 ± 9.9 38.7 to 71.5	14.9 ± 8.5 1.5 to 28.5 *P ≤ 0.0001*	14.8 ± 9.9 −9.5 to 28.5 *P ≤ 0.0001*
mHHS	60.6 ± 12.7 34.1 to 79.1	89.1 ± 14.0 50.5 to 100.0	85.3 ± 17.8 45.1 to 100.0	28.3 ± 14.3 0 to 53.8 *P ≤ 0.0001*	24.7 ± 17.4 −25.3 to 53.8 *P ≤ 0.0001*

ADL = activities of daily living, HOOS = hip disability and osteoarthritis outcome score, iHOT-12 = International Hip Outcome Tool, Short Form, mHHS = modified Harris hip score, PRO = patient-reported outcome, PROMIS = Patient-Reported Outcome Information System, PROMIS PF = Patient-Reported Outcome Measurement Information System Physical Function, VAS = visual analog scale

a Includes only those participants (n = 22/27) who completed both preoperative and postoperative testing, as described in Figure 2.

b *P* values indicate significance (*P* ≤ .05) as determined by Wilcoxon rank sum.

Values are expressed as mean ± SD, minimum and maximum.

**Table 3:** Minimal Clinically Important Difference (MCID) for Select PRO Measures

PRO Measure (MCID)	6 mo n/n (%)	12 mo n/n (%)
IHOT-12 (13-point increase)	17/19 (89.5)	20/22 (90.9)
HOOS pain (10-point increase)	18/19 (94.7)	20/22 (90.9)
HOOS PS (10-point increase)	17/19 (89.5)	20/22 (90.9)
mHHS (7.5-point increase)	17/19 (89.5)	19/22 (86.3)

HOOS = hip disability osteoarthritis outcome score, mHHS = modified Harris hip score, PRO = patient-reported outcome

### Physical Performance Measures

At 6 months post-PAO, the mean times for STS5 improved significantly (*P* = 0.020, Wilcoxon rank sum; Table 4). At 12 months, improvements in STS5 were maintained (*P* = 0.01), and TSA additionally demonstrated significant improvement (*P* = 0.03). Changes in FSST and SSWS did not reach significance (*P* = 0.07 and 0.08 at 6 months and 1 year, respectively). With the generalized linear modeling approach accounting for variation in the time to follow-up, the effect of PAO on STS5 was significant at both six months and one year and on TSA at 1 year (Supplemental Table 2, http://links.lww.com/JG9/A139).

**Table 4:** Physical Performance Measures

Cohort	5STS (s)	FSST (s)	SSWS (m/s)	TSA (s)
Healthy control subjects^a^ Scott et al^b^	5.9 ± 1.1 4.2-9.2 (0.9)	4.0 ± 0.6 3.1-5.5 (0.9)	1.5 ± 0.2 1.2-2.0 (1.5)	3.2 ± 0.3 2.6-3.8 (0.3)
Hip dysplasia n = 22^a^	10.2 ± 4.2 4.9-22.2 (4.0)	6.0 ± 1.8 4.0-10.0 (2.6)	1.2 ± 0.2 0.8-1.6 (0.4)	4.0 ± 1.0 2.5-6.4 (1.2)
6 mo post-PAO^a^ n = 19	8.3 ± 2.0 4.9-13.8 (2.1)	6.1 ± 1.5 3.8-9.1 (2.1)	1.3 ± 0.2 1.0-1.8 (0.4)	3.8 ± 0.6 2.7-5.0 (1.7)
12 mo post-PAO^a^ n = 22	8.40 ± 2.1 5.3-14.7 (2.4)	6.5 ± 1.6 4.2-12.6 (0.9)`	1.3 ± 0.2 0.9-1.8 (0.2)	3.70 ± 0.8 2.9-7.0 (0.9)
Δ pre-op to 6 mo^c^	−1.5 ± 2.3 −2.4-0.1 ***P =* 0.02**	−0.1 ± 1.6 −4.3-2.4 *P =* 0.93	+0.1 ± 0.2 −0.4-0.6 *P =* 0.26	−0.1 ± 0.6 −0.8-1.7 *P* = 0.47
Δ pre-op to 12 mo^c^	−1.8 ± 2.9 −8.6-1.5 ***P =* 0.01**	+0.5 ± 1.4 −2.7-3.0 *P* = 0.07	+0.1 ± 0.2 −0.2-0.3 *P* = 0.08	−0.3 ± 0.6 −0.6-2.0 ***P* = 0.03**
Δ 6–12 mo, *P* value	NS	NS	NS	NS

5STS = sit-to-stand 5 times test, ADL = activities of daily living, FSST = four-square-step test, NS = not significant, PAO = periacetabular osteotomy, SSWS = self-selected walking speed, TSA = timed stair ascent

a Values are expressed as mean ± SD and minimum and maximum (IQR).

b Previously published data, Scott et al^22^ OJSM 2020.

c Indicates significance (*P* ≤ 0.05) determined by Wilcoxon rank sum; (*P* > 0.05)

### Physical Performance Measure and Patient-reported Outcome Correlations

Of the four PPMs, the STS5 and TSA tests demonstrated the strongest correlation with PRO measures (Table 5). At 1 year, a statistically significant moderate to very strong monotonic correlation (ǀrǀ_s_ = 0.57 to 0.76, n = 22, *P* < 0.01) between STS5 and all PRO measures and their subscales (VAS, HOOS, iHOT-12, mHHS, PROMIS PI, and PROMIS PF) and a moderate to strong correlation (ǀrǀ_s_ = 0.42 to 0.58, *P* < 0.05) between TSA and all PROs except mHHS Pain were observed. At the preoperative and 6-month time points, STS5 and TSA tests also demonstrated moderate to strong correlations with most PRO measures (Table 5). A consistent trend was noted for physical function-specific PRO measures (mHHS function, gait and ADL, PROMIS PF, HOOS PS, and iHOT-12) to correlate more strongly with PPMs than pain-specific PRO subscales (VAS, mHHS Pain, HOOS Pain, and PROMIS PI). FSST did demonstrate some significant weak to moderate correlation (ǀrǀ_s_ = 0.36 to 0.58, *P* < 0.05) with function-based metrics at the preoperative time point (Table 5). SSWS did not markedly correlate with PROs at any time point.

**Table 5:** Spearman Correlation Coefficients for the Four Physical Performance Measures and Patient-Reported Outcome Measures at Each Time Point

PRO Measures	Preoperative n = 28^a^				6 mo After PAO n = 19				1 yr After PAO n = 22			
	STS5	FSST	SSWS	TSA	STS5	FSST	SSWS	TSA	STS5	FSST	SSWS	TSA
VAS	NS	NS	NS	NS	NS	NS	NS	NS	**0.712** **0.0002**	NS	NS	**0.425** **0.048**
HOOS pain	**−0.423** **0.0204**	NS	NS	NS	**−0.552** **0.021**	NS	NS	**−0.459** **0.063**	**−0.756** **<0.0001**	NS	NS	**−0.438** **0.041**
HOOS PS	**0.341** **0.075**	NS	NS	NS	**0.546** **0.023**	NS	NS	**0.437** **0.079**	**−0.706** **0.0002**	NS	NS	**−0.463** **0.029**
iHOT-12	**−0.605** **0.0006**	**−0.357** **0.062**	NS	**−0.461** **0.013**	**−0.629** **0.006**	NS	NS	**NS**	**−0.609** **0.0003**	NS	NS	**−0.417** **0.053**
PROMIS PF	**−0.646** **0.0002**	**−0.514** **0.005**	NS	**−0.559** **0.001**	**−0.692** **0.002**	NS	NS	**−0.581** **0.014**	**−0.648** **0.001**	NS	NS	**−0.584** **0.004**
PROMIS PI	**0.588** **0.001**	**0.428** **0.022**	NS	**0.521** **0.004**	**0.687** **0.002**	NS	NS	**0.545** **0.023**	**0.677** **0.0005**	NS	NS	**0.435** **0.043**
mHHS	**−0.603** **0.0007**	**−0.452** **0.0155**	NS	**−0.524** **0.004**	**−0.611** **0.009**	NS	NS	**−0.426** **0.087**	**−0.704** **0.0003**	NS	NS	**−0.455** **0.033**
MHHS ADLs	**−0.517** **0.004**	**−0.374** **0.049**	NS	**−0.463** **0.012**	**−0.469** **0.057**	NS	NS	**−0.476** **0.053**	**−0.681** **0.0005**	NS	NS	**−0.549** **0.008**
mHHS pain	NS	NS	NS	NS	**−0.539** **0.025**	NS	NS	NS	**−0.635** **0.0015**	NS	NS	NS
mHHS gait	**−0.584** **0.0011**	**−0.581** **0.001**	NS	**−0.659** **0.0001**	**−0.427** **0.086**	NS	NS	**−0.504** **0.039**	**−0.567** **0.0058**	NS	NS	**−0.476** **0.024**
mHHS function	**−0.630** **0.0003**	**−0.575** **0.0013**	NS	**−0.662** **0.0001**	**−0.570** **0.016**	NS	NS	**−0.581** **0.014**	**−0.678** **0.0005**	NS	NS	**−0.529** **0.011**

FSST = four-square-step test, HOOS = hip disability osteoarthritis outcome score, iHOT = International Hip Outcome Tool, mHHS = modified Harris hip score, NS = not significant, PAO = periacetabular osteotomy, PRO = patient-reported outcome, PROMIS PF = Patient-Reported Outcome Measurement Information System Physical Function, STS5 = sit-to-stand five times, SSWS, self-selected walking speed, TSA = timed stair ascent, VAS = visual analog scale

a n = 28 includes 29 participants who completed preoperative testing, with the one excluded who had incomplete PRO data.

The postoperative analyses included only those subjects (n = 19 and n = 22) who fully completed both preoperative and postoperative testings.

Data reported as R, *P* value (probability >|r| under H_0_); Rho = 0. NA meaning *P* > 0.05.

When participants who underwent contralateral hip surgical procedure during the study period were removed and those with unilateral dysplasia (n = 11) evaluated in isolation, correlations were noted to be substantially stronger at the 1-year time point (Supplemental Table 3, http://links.lww.com/JG9/A140), with ǀrǀ > 0.90, *P* < 0.001, for STS5 and TSA with multiple PROs and ǀrǀ > 0.64 to 0.71, *P* < 0.03, for FSST with several PRO measures as well.

### Patient Surveys

All participants (100%) found PPM testing acceptable to perform. During the follow-up, participants selected TSA as the most helpful test for gauging improvement (n = 17/19 and 18/22 at 6 and 12 months, respectively), followed by STS5 (n = 10/19 and 14/22, respectively), FSST (n = 8/19 and n = 11/22, respectively), and SSWS (n = 7/19 and n = 10/22, respectively). Four participants felt that PPM testing was more useful to them than PRO instruments, and 13 participants found PPMs and PRO instruments equally useful. No participants preferred traditional PRO testing to PPMs. Optional written feedback was uniformly positive; one subject at six months stated, “I feel like my performance has gotten better. It makes me feel like I made the right decision about surgery.”

## Discussion

This study evaluated the responsiveness of four PPMs (STS5, TSA, FSST, and SSWS) and their correlation with hip-specific PRO measures preoperatively and at 6 months and 1 year after PAO surgery. Our hypothesis that all four tests would show marked improvement at 6 months and 1 year was partially supported because, although all four tests detected dysfunction at baseline, only the STS5 and TSA tests demonstrated the ability to detect functional improvement post-PAO and statistically significant moderate to very strong correlation with PRO tests. The hypothesis that subjects would find PPM testing acceptable was also supported (100%), with no participants preferring standardized PRO testing to the novel PPM tests and some (n = 4) even preferring the PPM tests despite the physical nature of testing. Overall, our findings suggested that STS5 and TSA are responsive to changes in function that occur secondary to PAO surgery. These tests may be useful adjuncts to PROs for evaluating functional deficit related to hip dysplasia and for following up individual improvement after PAO surgery. Participants reported physical function testing to be acceptable despite disability or discomfort and found them more or equally useful to PRO measures for assessing their functional progress.

PPMs for this study were identified based on the study by Sheean et al describing baseline deficits in young adult military participants with femoracetabular impingement (FAI).^1^ Of these four tests, only TSA and STS5 ultimately demonstrated responsiveness in our cohort. In our predominantly female cohort, mean walking speed before surgery (1.2 m/s) was slower than healthy control subjects in the FAI study by Sheehan et al^23^ (mean 1.31 m/s) and healthy control subjects of similar age and sex (mean 1.5 m/s). SSWS did not improve for our participants post-PAO and failed to correlate with any PRO measures, suggesting that a walking speed test does not sufficiently target the deficits associated with dysplasia. Similarly, FSST also failed to improve post-PAO, with mean test times remaining approximately 6.0 to 6.5 ± 1.4 to 1.6 seconds throughout the study duration. Although requiring some single leg balance, the hip is relatively extended during this test, which may explain the lack of responsiveness in our cohort. The two physical performance tests that performed well in our study, STS5 and TSA, were also the most physically demanding. These tests evaluate coordinated lower extremity strength and require rapid and repetitive hip flexion. On subjective survey, participants correctly perceived these two tests as being both challenging to perform and a useful gauge of their functional abilities even after surgery. Considering STS5 can be done in virtually any examination space (without need for a staircase) and correlated moderately to very highly with PRO measures preoperatively and postoperatively, it should be of value to the hip surgeon interested in tracking functional improvements after PAO.

Baseline deficits and improvements in both PROs and PPMs varied considerably on an individual level. PROs at all time points were in line with values previously published for PAO.^9,11,31^ The ANCHOR cohort reported a mean HOOS Pain improvement of 28.3 (95% confidence interval, 25.3-30.1) at an average of 3 to 5 years of follow-up in their 391 patients compared with our mean increase of 34.9 ± 22.2 points at one year. Older age, female sex, elevated BMI, and concomitant ipsilateral procedures were found in that study to be independent predictors of patient-reported outcomes. Our cohort at one year had a similar mean age (25.5 ± 9.1 years, compared with 25.4 ± 9.5 years in the ANCHOR cohort) and similar BMI (24.6 ± versus 24.9 kg/m^2^; however, our study had a greater proportion of female subjects (91% versus 79%). Most patients (81%) of our cohort also had concomitant arthroscopy (percentage not reported in the ANCHOR study); these differences may explain the greater mean improvement we observed in PROs post-PAO. There were three participants who did not achieve MCID in PRO measures; interestingly, all three had bilateral hip dysplasia, with pain also in the contralateral hip. At one year, one participant was continuing to experience dysfunction related to their second PAO surgery. The other two participants were noted to be among the oldest participants in our cohort (aged 37 and 39 years) with Tönnis grade 1 hips on preoperative evaluation; these hips were examined arthroscopically at the time of PAO with evidence of labral damage with cartilage fissuring at the chondrolabral junction, likely, overall, indicating a more advanced level of hip degeneration.

Regarding the effect of bilateral disease, correlations between PRO measures and PPMs strengthened when evaluating only those with unilateral dysplasia (N = 11, Supplemental Table 3, http://links.lww.com/JG9/A140). Half (n = 11) of our cohort had bilateral dysplasia at the time of enrollment, and seven of these 11 participants underwent contralateral PAO and/or arthroscopy between six months and one year after their first PAO. We hypothesized that one might expect a greater functional deficit at baseline in participants with bilateral disease compared with those with a single affected hip and either a larger or smaller functional improvement depending on whether the contralateral hip was also treated. Proximity of surgery on the contralateral hip must also be taken into consideration when evaluating hip function in this cohort. Future studies with larger sample sizes of both unilateral and bilateral hips may identify significant functional differences between unilateral and bilateral disease and even ideal timing for treatment of the second hip. Our small sample size, loss to follow-up, and dropout after study initiation likely affected our ability to fully evaluate correlation between PPMs and PRO measures.

A primary limitation to this study was the small sample size. PPMs require in-person data collection, which limited our ability to enroll participants who would not follow up in person up to one year because of the long travel distance to our clinic. We also lost three participants to follow-up although 81% returned for PPM testing one year after surgery. The reasons for failure of follow-up included cancellation of visits for COVID-19 (1), prolonged medical illness (1), and relocating for school (1). Another limitation is the homogenous nature of the patient cohort we evaluated; although reflective of the local population in our area, it may limit the generalizability of our results to other more diverse populations.

In conclusion, we recommend use of the STS5 and TSA physical performance tests, for both preoperative evaluation and monitoring of functional improvement after PAO. At 6 months and 1 year after surgery, these tests correlated moderate to very strongly with common hip-specific PRO measures and provided an objective means of assessing disability that was both appealing to patients and easily performed without specialized equipment.

## Supplementary Material

SUPPLEMENTARY MATERIAL
