# Supplementary material for: Physical Performance Tests Correlate With Patient-reported Outcomes After Periacetabular Osteotomy: A Prospective Study
Source: J Am Acad Orthop Surg Glob Res Rev. 2021 Jun 8;5(6):e21.00100. doi: 10.5435/JAAOSGlobal-D-21-00100 (PMC8189615; doi:10.5435/JAAOSGlobal-D-21-00100)
Supplement: SUPPLEMENTARY MATERIAL [file jagrr-5-e21.00100-s001.docx]

**Supplemental Table 1.** PRO outcomes evaluated with generalized linear modeling with repeated measures. P-values were adjusted for multiple comparisons.

| **PROMIS PF** | | | | | | |
| --- | --- | --- | --- | --- | --- | --- |
| **Effect** | **Visit** | **Estimate** | **Standard Error** | **Lower** | **Upper** | **p-value:**  **compared with baseline** |
| **Visit** | **Avg 6 Months** | 52.35 | 1.88 | 48.67 | 56.02 | <0.0001 |
| **Visit** | **Avg 1 Year** | 52.50 | 2.08 | 48.42 | 56.57 | <0.0001 |
| **Visit** | **Baseline** | 41.34 | 1.30 | 38.79 | 43.88 | - |
| **PROMIS PI** | | | | | | |
| **Visit** | **Avg 6 Months** | 46.59 | 1.81 | 42.94 | 50.24 | <0.0001 |
| **Visit** | **Avg 1 Year** | 46.94 | 2.06 | 42.89 | 50.98 | <0.0001 |
| **Visit** | **Baseline** | 61.77 | 0.95 | 59.91 | 63.62 | - |
| **IHOT** | | | | | | |
| **Visit** | **Avg 6 Months** | 80.70 | 4.59 | 71.71 | 89.70 | <0.0001 |
| **Visit** | **Avg 1 Year** | 76.16 | 5.72 | 64.94 | 87.37 | <0.0001 |
| **Visit** | **Baseline** | 31.86 | 2.65 | 26.66 | 37.06 | - |
| **HOOS Pain** | | | | | | |
| **Visit** | **Avg 6 Months** | 85.66 | 3.79 | 78.23 | 93.09 | <0.0001 |
| **Visit** | **Avg 1 Year** | 82.27 | 5.08 | 72.31 | 92.23 | <0.0001 |
| **Visit** | **Baseline** | 47.41 | 2.98 | 41.57 | 53.24 | - |
| **HOOS PS** | | | | | | |
| **Visit** | **Avg 6 Months** | 81.81 | 5.20 | 71.61 | 92.01 | <0.0001 |
| **Visit** | **Avg 1 Year** | 78.84 | 5.51 | 68.05 | 89.63 | <0.0001 |
| **Visit** | **Baseline** | 38.53 | 3.28 | 32.11 | 44.95 | - |
| **MHHS** | | | | | | |
| **Visit** | **Avg 6 Months** | 81.05 | 2.84 | 75.49 | 86.62 | <0.0001 |
| **Visit** | **Avg 1 Year** | 77.59 | 3.38 | 70.97 | 84.21 | <0.0001 |
| **Visit** | **Baseline** | 55.14 | 2.41 | 50.42 | 59.86 | - |
| **VAS** | | | | | | |
| **Visit** | **Avg 6 Months** | 11.42 | 3.45 | 4.67 | 18.17 | <0.0001 |
| **Visit** | **Avg 1 Year** | 19.50 | 5.35 | 9.01 | 29.99 | <0.0001 |
| **Visit** | **Baseline** | 55.18 | 4.54 | 46.28 | 64.08 | - |
