# Supplementary material for: Physical Performance Tests Correlate With Patient-reported Outcomes After Periacetabular Osteotomy: A Prospective Study
Source: J Am Acad Orthop Surg Glob Res Rev. 2021 Jun 8;5(6):e21.00100. doi: 10.5435/JAAOSGlobal-D-21-00100 (PMC8189615; doi:10.5435/JAAOSGlobal-D-21-00100)
Supplement: SUPPLEMENTARY MATERIAL [file jagrr-5-e21.00100-s002.docx]

**Supplemental Table 2.** Generalized linear modeling with repeated measures evaluating the effect of PAO on physical performance measures. P-values were adjusted for multiple comparisons.

| **TSA** | | | | | |
| --- | --- | --- | --- | --- | --- |
| **Visit** | **Estimate** | **SE** | **Lower** | **Upper** | **p-value (compared with baseline)** |
| **Avg 6 Months** | 3.782 | 0.136 | 3.514 | 4.049 | 0.428 |
| **Avg 1 Year** | 3.657 | 0.171 | 3.321 | 3.992 | **0.037** |
| **Baseline** | 3.987 | 0.209 | 3.576 | 4.397 | - |
| **STS** | | | | | |
| **Avg 6 Months** | 8.311 | 0.449 | 7.430 | 9.193 | **0.032** |
| **Avg 1 Year** | 8.407 | 0.438 | 7.548 | 9.266 | **0.010** |
| **Baseline** | 10.171 | 0.867 | 8.470 | 11.871 | - |
| **FSST** | | | | | |
| **Avg 6 Months** | 6.069 | 0.346 | 5.391 | 6.748 | 0.957 |
| **Avg 1 Year** | 6.488 | 0.340 | 5.820 | 7.156 | 0.172 |
| **Baseline** | 5.964 | 0.380 | 5.218 | 6.711 | - |
| **SSWS** | | | | | |
| **Avg 6 Months** | 1.298 | 0.0509 | 1.198 | 1.397 | 0.228 |
| **Avg 1 Year** | 1.271 | 0.0424 | 1.188 | 1.354 | 0.200 |
| **Baseline** | 1.213 | 0.0471 | 1.121 | 1.306 | - |
