# Supplementary material for: Physical Performance Tests Correlate With Patient-reported Outcomes After Periacetabular Osteotomy: A Prospective Study
Source: J Am Acad Orthop Surg Glob Res Rev. 2021 Jun 8;5(6):e21.00100. doi: 10.5435/JAAOSGlobal-D-21-00100 (PMC8189615; doi:10.5435/JAAOSGlobal-D-21-00100)
Supplement: SUPPLEMENTARY MATERIAL [file jagrr-5-e21.00100-s003.docx]

**Supplemental Table 3.** Spearman Correlation Coefficients for the four PPMs and PROs at each timepoint for patients with unilateral disease (N=11).

|  | **Pre-Operative**  **N=11** | | | | **6 Months after PAO**  **N=8** | | | | **1 Year after PAO**  **N=11** | | | |
| --- | --- | --- | --- | --- | --- | --- | --- | --- | --- | --- | --- | --- |
|  | **STS5** | **FSST** | **SSWS** | **TSA** | **STS5** | **FSST** | **SSWS** | **TSA** | **STS5** | **FSST** | **SSWS** | **TSA** |
| VAS | NS | NS | NS | NS | NS | NS | NS | NS | **0.902**  **0.0001** | **0.646**  **0.031** | NS | **0.860**  **0.0007** |
| HOOS Pain | NS | NS | NS | NS | NS | NS | **-0.583**  **0.061** | NS | **-0.841**  **0.001** | **0.646**  **0.031** | NS | **-0.731**  **0.010** |
| HOOS PS | NS | NS | NS | NS | NS | NS | **-0.845**  **0.008** | NS | **-0.766**  **0.005** | NS | NS | **-0.915**  **<0.0001** |
| iHOT-12 | -**0.590**  **0.055** | NS | NS | **-0.681**  **0.020** | NS | NS | NS | NS | **-0.936**  **<.0001** | NS | NS | **-0.809**  **0.002** |
| PROMIS PF | NS | NS | NS | **-0.687**  **0.025** | **-0.687**  **0.059** | NS | NS | NS | **-0.750**  **0.007** | -**0.709**  **0.0145** | NS | **-0.855**  **0.0008** |
| PROMIS PI | **0.561**  **0.072** | **0.428**  **0.022** | NS | **0.667**  **0.025** | **0.672**  **0.067** | NS | NS | **0.735**  **0.037** | **0.738**  **0.009** | NS | NS | 0.644  0.032 |
| mHHS | NS | NS | NS | **-0.643**  **0.032** | **-0.675**  **0.065** | NS | NS | NS | **-0.698**  **0.016** | **-0.535**  **0.089** | NS | **-0.708**  **0.014** |
